# Short communication: relationships between novel breeding values for heat tolerance and rectal temperature during heat stress in lactating Holstein cows

**DOI:** 10.1093/jas/skaf386

**Published:** 2025-11-10

**Authors:** Serdal Dikmen, Natascha Vukasinovic, Miguel Angel Sánchez-Castro, Peter J Hansen

**Affiliations:** Department of Animal Science, Faculty of Veterinary Medicine, Bursa Uludag University, Bursa 16059, Türkiye; Department of Animal Sciences, University of Florida, Gainesville, FL 32611-0910; VMRD Genetics, Zoetis Inc, Kalamazoo, MI 49007; VMRD Genetics, Zoetis Inc, Kalamazoo, MI 49007; Department of Animal Sciences, University of Florida, Gainesville, FL 32611-0910

**Keywords:** Dairy cattle, heat stress, rectal temperature, resiliency, standardized transmitting abilities

## Abstract

Resistance of cows to heat stress is under genetic control. Recently, two novel breeding values for heat tolerance in lactating Holsteins have been developed based upon the change in conception at first service (CFS_THI) and milk yield (Milk_THI) with temperature-humidity index. The goal here was to test whether variation in standardized transmitting abilities (STA) for heat tolerance was associated with phenotypic variation in cow regulation of body temperature during heat stress. In this observational study, data on afternoon rectal temperatures (1400–1700 H) during the hot months of the year (May–September) were collected from lactating cows on farms in Florida (*n* = 3), California (*n* = 1), and Türkiye (*n* = 1) over a period spanning 2007 to 2023. Analyses were performed using records where the dry bulb temperature at cow side was ≥ 25 °C and STA reliability > 0.05. Data on 4,674 and 4,678 values of rectal temperature from 3,617 and 3,620 cows were analyzed for CFS_THI and Milk_THI, respectively. Standardized transmitting abilities were analyzed two ways—by comparing rectal temperatures of cows above and below a STA of 100 and by comparing cows in each quartile of the dataset. Rectal temperature was lower for cows with higher STA for CFS_THI but not for cows with higher STA for Milk_THI. Another analysis considered the percent of rectal temperatures > 39 °C (i.e., a body temperature more likely to result in compromised physiology and health). Higher STA for both CFS_THI and Milk_THI were associated with reduced percent rectal temperatures > 39 °C. In conclusion, results of this study demonstrate the link between genetic resistance to heat stress with respect to maintenance of fertility and milk yield with ability to regulate body temperature during heat stress. There is some evidence that this link is greater for effects of heat stress on fertility than on milk yield.

## Introduction

Heat stress is one of the most impactful environmental determinants of productivity and health of cattle, with adverse effects on milk production, sexual behavior, male and female fertility and fetal development (reviewed by Hansen, 2020). The degree to which heat stress compromises physiological function is under genetic control. Not only do breeds differ in responses to heat stress (Hansen, 2020) but there is genetic variation in heat tolerance within breeds of cattle that developed in temperate climates. The heritability of rectal temperature during heat stress in Holsteins was estimated at 0.17 (Dikmen et al., 2012), while the heritability of heat tolerance with respect to milk yield has been estimated to range between 0.09 and 0.32 (Sánchez et al., 2009; Bernabucci et al., 2014; Nguyen et al., 2016; Cheruiyot et al., 2022). Thus, genetic selection to reduce the impact of heat stress on dairy cattle should be possible.

An important determinant of the magnitude of heat stress effects on dairy cattle is the magnitude of the increase in hyperthermia arising from failure of cows to successfully regulate body temperature under hot environmental conditions. The reduction in milk yield (Umphrey et al., 2001; Igono and Johnson, 1990; Anzures-Olvera et al., 2019) and depression in fertility ­(Gwazdauskas et al., 1973) is proportional to the magnitude or duration of hyperthermia. It is likely, therefore, that cattle that are more genetically resistant to heat stress as measured by the change in milk yield or fertility with increasing heat stress will have lower rectal temperatures than cattle that are less genetically resistant. This was shown to be the case for a breeding value for thermotolerance based on the decline in milk yield per unit of temperature-humidity index (THI) increase derived for Holstein cattle in Australia (Garner et al., 2016; Jensen et al., 2022).

For the current study, we evaluated the ability of two new genomic breeding values of heat tolerance in dairy cattle developed by Zoetis Genetics to identify cows with superior ability to regulate body temperature during heat stress (Vukasinovic et al., 2025). One breeding value is based on the decline in cow conception rate with increasing THI (CFS_THI), whereas the other is based on the decrease in milk yield per unit increase in THI (MILK_THI). Results reported here indicate that both breeding values are associated with rectal temperature during heat stress.

## Materials and Methods

All procedures were approved by the Animal Care and Use Committee at the University of Florida and were performed in compliance with the guidelines and regulations. The study was conducted on lactating Holstein cows reared at 5 different dairies: University of Florida Dairy Unit (Hague, FL; 29°46′N 82°24′W), North Florida Holsteins (Bell, FL; 29°72′N 82°85′W), Southern Cross Dairy (O’Brien, FL; 30°08′N 83°02′W), Maddox Dairy (Riverdale, CA; 36°53′N 120°02′W) and Ozlem Dairy (Manisa, Turkiye; 38°58′N 28°12′E). Except for North Florida Holsteins, which utilized tunnel ventilation for cooling cows, cows at each farm were housed in freestall barns equipped with fans and sprinklers. Cows at each farm were fed total mixed rations and had free access to water.

Rectal temperature data were collected between 2007 to 2023 during the hottest months of the year (May to September) and at critical hours of exposure to heat stress (between 1400 and 1700 h). Measurements were taken while cows were resting in freestalls, without any kind of management practice (excessive walking or physical restriction) that could alter their temperature response. Observations in US farms were obtained using a digital GLA M750 thermometer with ± 0.01 accuracy (GLA Agricultural Electronics, San Luis Obispo, CA), while for the Turkish dairy a Kerbl TopTemp digital thermometer with ± 0.01 accuracy (ENKA Tarim Hayvancilik San. Tic. Ltd Şti. Izmir, Türkiye) was utilized. In general, each cow was measured an average of 1.34 times (range 1–8) and some of these records have been used in earlier studies (Dikmen and Hansen, 2009; Dikmen et al., 2020; Jensen et al., 2022).

Measurements of dry bulb temperature (Tdb), relative humidity (RH), dew point temperature (Tdp), and black globe temperature (Tbg) were measured at 1-min intervals between 1400 and 1700 h using a Hobo-U12 data logger (Tdb, RH, and Tdp) and a Hobo Water Temp Pro V2 data logger (Tbg) (Onset Computer Corp., Bourne, MA) that were located at a height of 2 m from the ground at a position in the center of the barn where cows were housed. Rectal temperature was matched with the measurements of Tdb, RH, Tdp, and Tbg to the nearest minute at which environmental variables were recorded.

Temperature-humidity index (THI) was calculated as follows (National Research Council, NRC, 1971):


THI=1.8×Tdb+32−[0.55−0.0055×RH ×1.8×Tdb−26.8],

where Tdb = dry bulb temperature (°C) and RH = relative humidity.

DNA collected from hair, blood or ear punch was genotyped with the Illumina 50 K BeadChip (Zoetis, Kalamazoo, MI, USA). Genomic breeding values for CFS_THI and Milk_THI were calculated by Zoetis Genetics based on methodology described by Vukasinovic et al. (2025). In brief, test-day milk yields and insemination records from 370 herds across 30 states from the years 2001–2021 were merged with THI weather data. About 83 million test day milk records and over 6 million inseminations were available for analysis. The effect of heat stress was modeled using a bivariate reaction norm linear model that assumed the negative impact of heat stress occurred at THI ≥ 70. The evaluation was conducted using the single-step genomic BLUP (ssGBLUP) methodology, applying the algorithm for proven and young animals. Over 2 million genotyped animals were available. The model for conception at first service (CFS) included herd-year-season of calving, parity, breeding type, and voluntary waiting period as fixed effects while the additive genetic effect, the random regression on THI for heat stress genetic effect, the permanent environment, and the random regression on THI for the permanent environment effect were considered random. The model for milk yield included herd-year-season, parity, and days-in-milk (DIM) classes as fixed effects and the same random effects as described for CFS. Given each trait had a different average and range of Predicted Transmitting Ability (PTA), the PTA obtained for both traits were linearly transformed and expressed as genomic Standardized Transmitting Abilities (STA), with a mean of 100 and a standard deviation of 5. Within this transformation, higher values indicate better heat tolerance. Additionally, it is important to highlight that none of the animals in this study contributed phenotypes (cow conception rate or milk yield) to the genomic evaluation; therefore, their breeding values were obtained using only genomic data and/or pedigree information contained in the H matrix used on the ssGBLUP method.

Effects of heat tolerance breeding values on rectal temperature were analyzed by analysis of variance with the GLIMMIX procedure of the Statistical Analysis System (SAS) v 9.4 (SAS Institute, Cary, NC, USA). The statistical model used in this study is given below:


$$Y_{\text{ijklm}}=\mu +{\;a}_{i}+{\;b}_{j}+{\;c}_{k}+{\left(\text{da}\right)}_{\text{li}}+{\left(\text{db}\right)}_{\text{lj}}+{\;f}_{m}+{\;e}_{\text{ijklm}}$$

Where Y_ijklm_ = rectal temperature, μ= population average, a_i_ = ith parity effect (*i* = 1, 2, ≥3), *b_j_* = *j*th stage of lactation (*j* = stage of lactation < 100 DIM, 101–200 DIM, and > 200 DIM), *c_k_* = *k*th year-farm effect, (da)_li_ = effect of STA class (classes defined later in text) and parity interaction, (db)_lj_ = effect of STA class and stage of lactation interaction, *f*_m_= random effect of cow, *e*_ijklm_ = is the residual error.

Rectal temperature data included in the analyses were from records where the Tdb at cowside was ≥ 25 °C and reliability of predicted transmitting ability for CFS_THI or MILK_THI was > 0.05. The minimum reliability value of 0.05 was chosen arbitrarily to minimize the number of animals excluded from the study and at the same time ensure that STAs were based on the maximum available information. After screening, the average reliability was 0.466 [standard deviation (SD) =0.124] for CFS_THI and 0.660 (SD = 0.143) for Milk_THI. For CFS_THI, although a total of 4,842 rectal temperatures were available, only 4,674 observations from 3,617 cows were used in the GLIMMIX statistical analysis (cows in which there was a missing observation for one term in the model were excluded). Similarly, for MILK_THI although a total of 4,846 rectal temperatures were available, just 4,678 records from 3,620 cows were used in the statistical analysis. For both variables, the mean rectal temperature was 38.68 °C (SD = 0.61 °C) and the mean THI was 82.09 units (SD = 3.10 units).

Standardized transmitting abilities were analyzed two ways—by comparing rectal temperatures of cows with a STA ≤ 100 or > 100 and by comparing cows in each quartile of the STA distribution. Data were reanalyzed after removing interaction terms that were not significant (*P* > 0.05). When a main effect with more than 1 degree of freedom was statistically significant (*P* < 0.05), differences among individual means were determined by calulating pairwise t tests using the PDIFF procedure of SAS with a Bonferroni adjustment for multiple testing.

Chi-square analysis was used to determine effects of STA for heat tolerance on the distribution of rectal temperatures into the categories of > 39 °C vs ≤ 39°C. For this analysis, all the data originally available was used (*n* = 4,842 for CFS_THI and 4,864 for Milk_THI). Based on analysis of distribution of rectal temperatures of cows experiencing THI in the thermoneutral range (Dikmen and Hansen, 2009), the value of 39 °C was considered a rectal temperature that would exclude most cows that were successfully regulating body temperature. It was assumed, therefore, that cows experiencing rectal temperatures above 39°C would be at increased risk to compromised physiology and health.

## Results and Discussion

**Figure 1.**
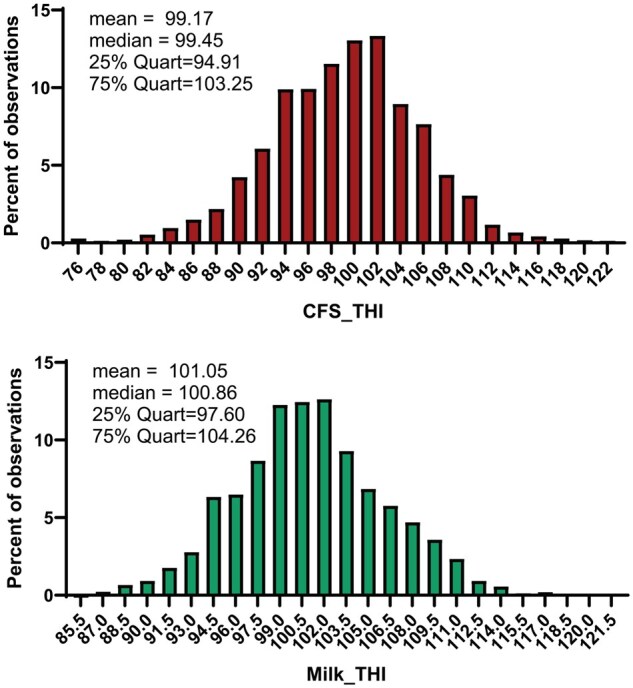
Distribution of STA for CFS_THI (*n* = 3,620 cows, top) and MILK_THI (*n* = 3,617 cows, bottom) of lactating Holstein cows located in 5 different farms in the USA and Türkiye from 2007 to 2023.

**Figure 2.**
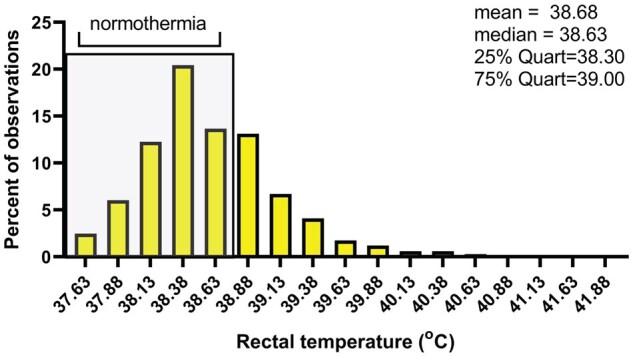
Distribution of 4,846 recordings of rectal temperatures from 3,620 lactating Holstein cows located in 5 different farms in the USA and Türkiye from 2007 to 2023. Temperatures characteristic of those under normothermia are marked by shading.

Distribution of data for STA for CFS_THI and MILK_THI are shown in Figure 1. The mean value for CFS_THI was 99.17 and the median was 99.45. The mean value for MILK_THI was 101.05 and the median was 100.86. Distribution of rectal temperatures for cows are shown in Figure 2. The mean rectal temperature was 38.68 °C and the median was 38.63 °C. Normal body temperature of cows in the absence of heat stress ranges between 38.3 and 38.6 °C (Rungruang et al., 2014). Thus, only about 50% of cows experienced rectal temperatures above this range. Only 25% of cows experienced rectal temperatures greater than 39 °C (i.e., the 3^rd^ quartile of rectal temperatures).

In the first analysis, cows with a STA ≤ 100 were described as heat-sensitive and those with a STA > 100 were considered as heat-tolerant. Heat-sensitive cows based on CFS_THI STA had higher (*P* < 0.0001) rectal temperatures than cows classified as heat-tolerant (38.70 ± 0.01 vs 38.62 ± 0.01 °C). There was a tendency (*P* = 0.10) for an interaction between parity and STA class because differences between heat-sensitive and heat-tolerant cows was greater for parity 1 (38.69 ± 0.02 vs 38.58 ± 0.02 °C) and 2 (38.70 ± 0.03 vs 38.59 ± 0.03 °C) than for cows that were parity 3 or greater (38.72 ± 0.02 vs 38.70 ± 0.03 °C). The percent of animals with rectal temperatures >39°C within the heat-sensitive and heat-tolerant groups was also analyzed. A significant difference (*P* < 0.0001) was detected depending on the heat tolerance classification. Specifically, 27.4% of the heat-sensitive cows (729/2663) had a rectal temperature >39°C, while 22.0% of the heat-tolerant cows (479/2179) were hyperthermic.

In contrast to the results obtained when classifying animals based on CFS_THI STA, there was no significant difference in rectal temperature (*P* = 0.7328) between heat-sensitive and heat-tolerant cows based on Milk_THI STA (38.67 ± 0.01 vs 38.66 ± 0.01 °C). There were also no interactions with stage of lactation or parity. Nonetheless, the percent of animals with rectal temperatures >39°C was higher (*P* = 0.0010) for heat-sensitive cows (27.1%; 611/2251) than for heat-tolerant cows (23.0%; 598/2595).

In the second analysis, differences in rectal temperature due to STA for heat tolerance were analyzed using quartile classes derived from CFS_THI and MILK_THI STA distributions. Results are shown in Table 1. Rectal temperature varied with CFS_THI quartile class (*P* = 0.0353) since the average rectal temperature was lower in quartile 4 (STA ≥ 103.25) than quartile 1 (STA ≤ 94.91) and quartile 2 (STA between 94.91 and 99.45). There was no effect on average rectal temperature detected for STA quartiles based on Milk_THI. The percent of rectal temperatures >39°C was affected by quartile class for both CFS_THI (*P* < 0.0001) and Milk_THI (*P* = 0.0009). Percent rectal temperatures >39°C was least for quartile 4, highest for quartile 1, with values for quartiles 2 and 3 being intermediate.

**Table 1.** Results of analysis of rectal temperatures based on quartiles of STA for CFS_THI and Milk_THI

CFS_THI			Milk_THI		
Quartile	Rectal temperature (°C)	Rectal temperatures > 39°C (%, *n*/*n*)	Quartile	Rectal temperature (°C)	Rectal temperatures > 39°C (%, *n*/*n*)
1 (≤94.91)	38.67 ± 0.06^a^	29.4% (383/1305)	1 (≤97.60)	38.63 ± 0.06^a^	28.0% (372/1328)
2 (>94.91 to <99.45)	38.65 ± 0.06^a^	25.3% (303/1196)	2 (>97.60 to <100.86)	38.65 ± 0.06^a^	26.0% (326/1253)
3 (99.45 to <103.25)	38.63 ± 0.06^ab^	24.1% (285/1183)	3 (100.86 to <104.26)	38.64 ± 0.06^a^	23.9% (282/1181)
4 (≥103.25)	38.60 ± 0.06^b^	20.5% (237/1158)	4 (≥104.26)	38.64 ± 0.06^a^	21.1% (229/1084)
*P*, quartile	0.0353	<0.0001		0.7514	0.0009

a,b Means with different subscripts differ (*P* < 0.05).

It is now apparent that genetic variation in resistance to heat stress exists within breeds like the Holstein that evolved in temperate climates (Sánchez et al., 2009; Dikmen et al., 2012; Bernabucci et al., 2014; Nguyen et al., 2016; Cheruiyot et al., 2022; Jensen et al., 2022). The breeding values for thermotolerance explored here were calculated based on the magnitude of change in either conception at first service (CFS_THI) or milk yield (Milk_THI) with increasing THI. Both breeding values (expressed as STA) were shown to be effective in distinguishing animals based on regulation of body temperature during heat stress, with the relationship between CFS_THI and rectal temperature being more consistently observed than the relationship between Milk_THI and rectal temperature.

One of the characteristics of the data set was that many cows were successful in maintaining normothermia even though the study was performed in regions of the world characterized by widespread and severe heat stress. All the farms studied made extensive use of cooling systems in cow housing which most likely reduced the magnitude of heat stress experienced by cows so that most cows could successfully thermoregulate. Moreover, most cows might have become physiologically adapted to heat stress. Only ∼50% of rectal temperatures were >38.6 °C and 25% were >39°C. One consequence was that the absolute magnitude of differences in mean rectal temperature between genetic classes was small. A similar result was observed when evaluating the relationship between breeding values for thermotolerance derived in Australia and phenotypic values for rectal and vaginal temperatures (Garner et al., 2016; Jensen et al., 2022). The fact that mean rectal temperatures differed by less than 0.1 °C between genetic classes does not mean that differences in body temperature regulation are insignificant. The magnitude of heat stress effects on milk yield (Igano and Johnson, 1990; Umphrey et al., 2001; Anzures-Olvera et al., 2019) and fertility (Gwazdauskas et al., 1973) are related to the degree of hyperthermia. It is notable that while 29.4% of cows in the lowest quartile of STA for CFS_THI experienced rectal temperatures >39°C, this was the case for only 20.5% of cows in the highest quartile. Similar observations were made for Milk_THI. This difference in the proportion of cows unable to prevent a large increase in body temperature during heat stress would be expected to translate into altered physiological and productive functions.

At least with respect to mean rectal temperature, CFS_THI was more closely related to resistance to heat stress than Milk_THI. One reason might be related to the physiological causes for the reduction in fertility and milk yield caused by heat stress. One major cause of infertility during heat stress is the direct effects of elevated temperature on the early preimplantation embryo (Hansen, 2019). Effects of heat stress on milk yield involve reductions in feed intake (McGuire et al., 1989; Wheelock et al., 2010) as well as alterations in endocrine function and change energy metabolism (Rhoads et al., 2010; Baumgard et al., 2011; Gao et al., 2017). It is possible that there are more genes associated with the magnitude of heat stress effects on milk yield than on fertility, for example those related to feed intake or metabolism. Indirect evidence for heat resistance genes independent of those controlling body temperature was obtained recently from an experiment comparing heat stress responses in Holstein and Brown Swiss cows ­(Cuellar et al., 2023). In that study, the magnitude of the seasonal depression in milk yield was similar for both breeds even though Brown Swiss were superior at regulation of vaginal temperature during heat stress. Results of genome-wide association studies are indicative that many of the genes associated with quantitative trait loci for heat tolerance with respect to milk yield are in genes that control cellular responses to elevated temperature (Sigdel et al., 2019; Cheruiyot et al., 2021).

In conclusion, results of this study demonstrate the link between genetic resistance to heat stress with respect to maintenance of fertility and milk yield with ability to regulate body temperature during heat stress. There is some evidence that this link is greater for effects of heat stress on fertility than on milk yield.

## Abbreviations:

CFS_THI: breeding value based on the decline in conception rate at first service with increasing temperature—humidity index
DIM: days in milk
MILK_THI: breeding value based on the decline in milk yield with increasing temperature—humidity index
PTA: predicted transmitting ability
RH: relative humidity
SD: standard deviation
ssGBLUP: single step genomic best linear unbiased prediction
STA: standard transmitting ability
Tdb: dry bulb temperature
THI: temperature-humidity index
